# Angiogenesis and hypoxia in lymph node metastases is predicted by the angiogenesis and hypoxia in the primary tumour in patients with breast cancer

**DOI:** 10.1038/sj.bjc.6602828

**Published:** 2005-10-25

**Authors:** G G Van den Eynden, I Van der Auwera, S J Van Laere, C G Colpaert, H Turley, A L Harris, P van Dam, L Y Dirix, P B Vermeulen, E A Van Marck

**Affiliations:** 1 Translational Cancer Research Group, Lab Pathology University of Antwerp/University Hospital Antwerp, Wilrijk, Antwerp B-2610, Belgium; 2 Translational Cancer Research Group, Oncology Center, General Hospital St-Augustinus, Wilrijk, Antwerp B-2610, Belgium; 3 Nuffield Department Clinical Laboratory Sciences, University of Oxford – John Radcliffe Hospital, Headley Way, Oxford, Oxfordshire OX3 9DU, UK; 4 Cancer Research UK Growth Factor Group, Weatherall Institute of Molecular Medicine, John Radcliffe Hospital, Oxford, Oxfordshire OX3 9DU, UK

**Keywords:** angiogenesis, hypoxia, lymph node metastasis, breast cancer

## Abstract

Hypoxia and angiogenesis are important factors in breast cancer progression. Little is known of hypoxia and angiogenesis in lymph node metastases of breast cancer. The aim of this study was to quantify hypoxia, by hypoxia-induced marker expression levels, and angiogenesis, by endothelial cell proliferation, comparing primary breast tumours and axillary lymph node metastases. Tissue sections of the primary tumour and a lymph node metastasis of 60 patients with breast cancer were immunohistochemically stained for the hypoxia-markers carbonic anhydrase 9 (CA9), hypoxia-inducible factor-1*α* (Hif-1*α*) and DEC-1 and for CD34/Ki-67. Endothelial cell proliferation fraction (ECP%) and tumour cell proliferation fraction (TCP%) were assessed. On haematoxylin–eosin stain, the growth pattern and the presence of a fibrotic focus were assessed. Hypoxia-marker expression, ECP% and TCP% in primary tumours and in lymph node metastases were correlated to each other and to clinico-pathological variables. Median ECP% and TCP% in primary tumours and lymph node metastases were comparable (primary tumours: ECP%=4.02, TCP%=19.54; lymph node metastases: ECP%=5.47, TCP%=21.26). ECP% correlated with TCP% (primary tumours: *r*=0.63, *P*<0.001; lymph node metastases: *r*=0.76, *P*<0.001). CA9 and Hif-1*α* expression were correlated (primary tumours *P*=0.005; lymph node metastases *P*<0.001). In primary tumours, CA9 and Hif-1*α* expression were correlated with DEC-1 expression (*P*=0.05), presence of a fibrotic focus (*P*<0.007) and mixed/expansive growth pattern (*P*<0.001). Primary tumours and lymph node metastases with CA9 or Hif-1*α* expression had a higher ECP% and TCP% (*P*<0.003); in primary tumours, mixed/expansive growth pattern and fibrotic focus were characterised by higher ECP% (*P*=0.03). Furthermore, between primary tumours and lymph node metastases a correlation was found for ECP%, TCP%, CA9 and Hif-1*α* expression (ECP% *r*=0.51, *P*<0.001; TCP *r*=0.77, *P*<0.001; CA9 and Hif-1*α P*<0.001). Our data demonstrate that the growth of breast cancer lymph node metastases is angiogenesis dependent and that angiogenesis and hypoxia in the primary tumour predict angiogenesis and hypoxia in the lymph node metastases. Together with previous findings in breast cancer liver metastases, which grow in 96% of cases angiogenesis independently, these data suggest that both the intrinsic growth characteristics and angiogenic potential of breast cancer cells and the site-specific tumour microenvironment determine angiogenesis and hypoxia in breast cancer.

Breast cancer is the most frequent neoplasm in women and the most frequent cause of death in women between 35 and 55 years of age (Uzzan *et al*, 2004). Although loco-regional spread and recurrence of the disease can be debilitating, metastasis to distant organs is the leading cause of breast cancer-related death. Traditional metastasis models of breast cancer include the regional lymph node basin as the first station in the metastatic cascade from where further haematogenous dissemination to distant organs occurs. In these models, the process of metastasis occurs in an orderly fashion from the primary tumour to the regional lymph nodes prior to systemic dissemination (Nathanson, 2003). Lymph node involvement indeed predicts the likelihood of survival as well as the likelihood that distant haematogenous metastases will develop (Donegan, 1992; Heimann and Hellman, 2000; Chang *et al*, 2003; Subramaniam and Isaacs, 2005). Nevertheless, recent data (reviewed by Pantel *et al*) have supported models in which haematogenous dissemination in breast cancer can either occur directly from the primary tumour or from metastases that develop in the lymph nodes or in distant organs (Pantel and Brakenhoff, 2004).

Little is known about the contribution of established lymph node metastases to the formation of blood-borne, distant metastases. Guidi *et al* (2000) demonstrated that the presence of vascular hotspots and increased microvessel density in lymph node metastases of breast cancer and not in the primary tumour were correlated with a worse prognosis. Their results suggest that angiogenesis in the lymph node metastases may contribute to disease progression and the haematogenous spread of tumour cells. Angiogenesis indeed plays an essential role in several aspects of neoplastic invasion and progression (Folkman, 1986). In breast cancer, angiogenesis in the primary tumour has been found to predict relapse-free survival and overall survival (Weidner *et al*, 1991; Horak *et al*, 1992; Fox *et al*, 1994; Gasparini *et al*, 1994, 1997; Linderholm *et al*, 2000).

Another important component of tumour progression, and a driving force of angiogenesis, is hypoxia (Chia *et al*, 2001; Schindl *et al*, 2002; Bos *et al*, 2003; Colpaert *et al*, 2003a; Dery *et al*, 2005). Under hypoxic conditions, hypoxia-inducible factor-1*α* (Hif-1*α*) is stabilized and serves to propagate a cascade of pathways which include angiogenesis, glycolysis, proliferation and alterations in microenvironmental pH (carbonic anhydrase 9 (CA9)) (Semenza, 1999). Differentiated embryo-chondrocyte-expressed gene 1 (DEC-1) or STRA13 is one of the transcription factors involved in cell differentiation, proliferation and apoptosis that has been shown to be Hif-1*α* regulated (Wykoff *et al*, 2000). The expression patterns of CA9, Hif-1*α* and DEC-1 have been shown to correlate with each other, with angiogenesis and with the expression of angiogenic factors (Giatromanolaki *et al*, 2003; Chakrabarti *et al*, 2004; Currie *et al*, 2004; Bos *et al*, 2005; Vleugel *et al*, 2005). The profound changes in different pathways due to hypoxia facilitate tumour progression and invasion and lead to resistance to chemo- and radiotherapeutic interventions (Harris, 2002; Vaupel, 2004).

Although primary tumour growth in breast cancer is angiogenesis dependent, we previously showed that 96% of liver metastases of breast cancer grow according to a nonangiogenic replacement pattern, marked by replacement of the hepatocytes by tumour cells without any desmoplastic reaction (Stessels *et al*, 2004). Thus, breast cancer liver metastases grow by coopting the sinusoidal vessels. Furthermore, 51% of breast cancer skin deposits grows according to a less angiogenic infiltrative pattern with lower microvessel density, endothelial cell proliferation fraction (ECP%) and CA9 expression (Colpaert *et al*, 2003b). Thus, the growth pattern and the degree of angiogenesis of breast cancer seems to be influenced by the local site-specific tumour microenvironment. Only limited and conflicting data on angiogenesis, and, to the best of our knowledge, no data on hypoxia in lymph node metastases of breast cancer are available (Arapandoni-Dadioti *et al*, 1999; Edel *et al*, 2000; Guidi *et al*, 2000). It is also of note that recent systemic therapy approaches to control cancer include inhibitors of the Hif pathway (Chau *et al*, 2005; Joyce, 2005; Tan *et al*, 2005), yet nearly all studies on Hif are on primary tumours and it is not known whether activation of Hif in the primary tumour is reflected at secondary sites. If hypoxia is caused by oxygen utilisation due to the particular oncogene pathway that is activated, as well as poor angiogenesis, it is possible that this will result in similar hypoxia at distant sites, whereas differences in angiogenesis response in the tissue, may result in substantial discrepancies.

Therefore, the aim of this study was to quantify hypoxia, by hypoxia-induced marker expression levels, and angiogenesis, by endothelial cell proliferation, comparing primary breast tumours and axillary lymph node metastases.

## MATERIALS AND METHODS

### Patient selection

In all, 60 newly diagnosed breast cancer patients – treated at the General Hospital St-Augustinus, Wilrijk, Belgium – with histopathological-confirmed lymph node involvement at time of diagnosis were included, after written informed consent. All protocols were reviewed and approved by the ethical committee of the General Hospital St-Augustinus. Metastases with complete replacement of the lymph node parenchyma by tumour tissue were not eligible. Furthermore, only patients that did not receive any breast cancer-related treatment (hormonal, chemo- and/or radiotherapy) prior to surgery were included in this study. Table 1 summarizes clinico-pathological data of the study population. Age, histological type, estrogen and progesteron receptor status, p53 and Her2/neu oncoprotein status were recorded by review of pathology files.

All haematoxylin–eosin (HE)-stained slides of the primary tumour and of the lymph nodes were reviewed to select one paraffin block of the primary tumour and one representative paraffin block of a lymph node metastasis, that is with tumour/normal tissue interface, for additional immunohistochemical (IHC) stains. If more than one lymph node metastasis was suitable for analyses, one lymph node metastasis was randomly chosen. In 15 cases, a paraffin block from a second lymph node metastasis was also selected. In four cases, lymph node metastases were too small to obtain enough slides for all the IHC stains planned, in one case the primary tumour could not be retrieved. On the HE slides, the growth pattern and the presence of a fibrotic focus in the primary tumour were assessed as previously defined (Hasebe *et al*, 1998; Colpaert *et al*, 2001, 2003b). In the infiltrative growth pattern, carcinoma cells infiltrate between pre-existing breast parenchymal structures, without significant disturbance of the breast architecture. In the expansive growth pattern, the tumour forms a well-circumscribed nodule consisting of carcinoma cells and desmoplastic connective tissue. Pre-existing breast parenchymal structures are not present inside the tumour but are pushed aside by the expansively growing nodule. The growth pattern is mixed infiltrative–expansive, when the tumour consists of a central expansive nodule surrounded by carcinoma cells showing an infiltrative growth pattern. A fibrotic focus is defined as a scar-like area, consisting of fibroblasts and collagen fibres, that occupies various percentages of the centre of an invasive ductal carcinoma of the breast. The size of the lymph node metastases was measured with a ruler.

### Endothelial and tumour cell proliferation fraction (TCP%)

**Figure 1:**
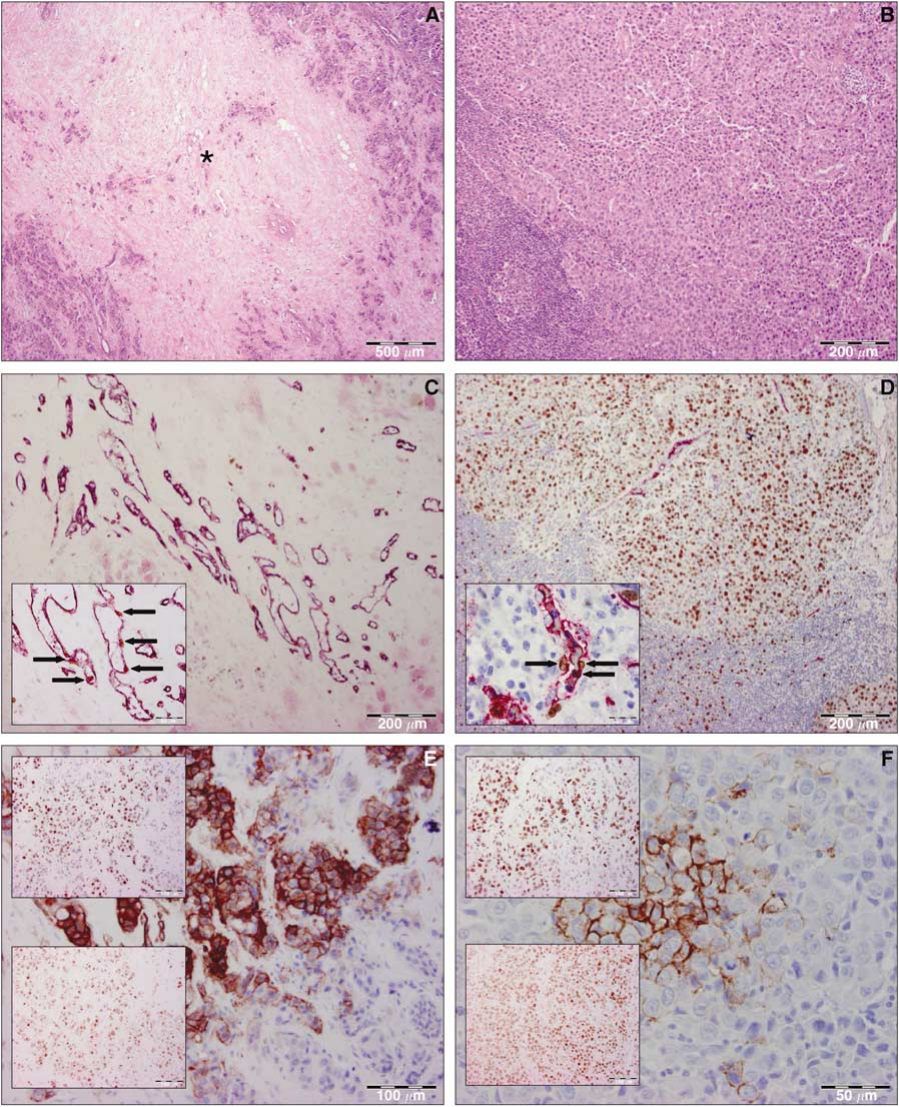
Overview of the histological and immunohistochemical stainings used, in a primary tumour (left: **A**, **C** and **E**) and in the corresponding lymph node metastasis (right: **B**, **D** and **F**) with expression of hypoxia markers and high ECP%. **A** and **B**: HE staining showed the presence of a large fibrotic focus (^*^) in the primary tumour. **C** and **D**: On CD34/Ki-67 IHC double-staining ECP% was high. Insets show Ki-67-positive (brown nucleus) endothelial cells (red cytoplasm) (black arrows). **E** and **F**: Membranous CA9 staining of tumour cells in both the primary tumour and in the lymph node metastasis was demonstrated. Insets show nuclear Hif-1*α* (upper inset) and DEC-1 (lower inset) expression in the same tumour region. ECP%: endothelial cell proliferation fraction; HE: haematoxylin–eosin; CA9: carbonic anhydrase 9; Hif-1*α*: hypoxia-inducible factor-1*α*; DEC-1: differentiated embryo-chondrocyte-expressed gene 1.

Figure 1 presents an overview of all histological and IHC stainings used in this study. For the visualisation of proliferating endothelial cells, a CD34/Ki67 double-stain procedure was performed on an automated IHC staining system (DakoCytomation Autostainer; DakoCytomation, Glostrüp, Denmark). This technique is used to simultaneously stain endothelial cells (cytoplasmic CD34) and proliferating cells (nuclear Ki-67). After rehydration of 5 *μ*m slides through a graded alcohol series, antigen retrieval was performed by heating the slides for 30 min in sodium-citrate buffer using a warm water bath at 98°C. Endogenous peroxidase was quenched for 10 min with 3% hydrogen peroxide (VWR International, Leuven, Belgium) and the anti-Ki-67 primary antibody (clone MIB-1, DakoCytomation, diluted 1/150) was incubated for 30 min followed by 30 min of the horseradish peroxidase-labelled polymer and 10 min of the DAB from the ChemMate Envision+detection system (DakoCytomation). After rinsing for 3 min in sodium-citrate buffer, sections were incubated with a second primary antibody directed against CD34 (clone Qbend 10, DakoCytomation, diluted 1/50) for 60 min followed by 30 min of alkaline phosphatase-labelled polymer and 15 min of fast red staining solution from the ChemMate Envision+detection system (DakoCytomation).

The fraction of proliferating endothelial cells (ECP%) and the fraction of proliferating tumour cells (TCP%) were assessed at the tumour/normal tissue interface, identified at low magnification (× 10 ocular and × 10 objective). On a higher magnification (× 10 ocular and × 40 objective) a total number of approximately 500 endothelial (=CD34 positive) or tumour cells, respectively, were evaluated on consecutive fields. ECP% and TCP% were calculated according to the following formulas : ECP%=(the number of endothelial cells with Ki67-stained nuclei/total number of endothelial cells evaluated) × 100; TCP%=(the number of tumour cells with Ki67-stained nuclei/total number of tumour cells evaluated) × 100. For statistical analysis, ECP% and TCP% in the primary tumour and in the lymph node metastases were also dichotomised using the median as a cutoff.

### Hypoxia

For the IHC evaluation of hypoxia, slides were stained for the following antigens: CA9, DEC-1 and Hif-1*α*. CA9 and DEC-1 IHC staining were automated using the DakoCytomation Autostainer (DakoCytomation). Primary antibodies anti-CA9 (Rabbit polyclonal, NB 100-417A, Novus Biologicals, Littleton, CO, USA, diluted 1/2000) and anti-DEC-1 (CW27 rabbit polyclonal, Kindly provided by Professor Dr A Harris, diluted 1/1000) were incubated for 60 min at room temperature. Antibody binding was visualised using the ChemMate Envision+detection system (DakoCytomation). Semiquantitative analysis of CA9 and DEC-1 expression was carried out as described before (Chia *et al*, 2001; Chakrabarti *et al*, 2004). In brief, for CA9 a score of 0–3 for intensity of staining was given (0: no staining, 1: weak staining, 2: moderate staining, 3: strong staining). The percentage of immunostained tumour cells was estimated. The product (intensity score × the percentage of immunoreactive tumour cells) yielded a final score of 0–300. For statistical evaluation, any CA9 score above 0 was called positive. For DEC-1, a 0–3 score was given based on intensity of nuclear staining of tumour cells (0: no staining, 1: weak staining, 2: moderate staining, 3: strong staining). For statistical analysis, DEC-1 score 0–1 was called negative, DEC-1 score 2–3 was called positive. The Hif-1*α* IHC staining was carried out manually, slightly modifying the protocol as described by Bos *et al* (2001). Briefly, slides were deparaffinised and rehydrated. After endogenous peroxidase blocking in 3% hydrogen peroxide (VWR International) and antigen retrieval using Target Retrieval Solution (S1699, DakoCytomation) and warm water bath at 98°C, the Catalysed Signal Amplification system (DakoCytomation) was used for Hif-1*α* staining according to the manufacturer's instructions. Anti-Hif-1*α* primary antibody (Clone 54, BD Pharmingen, Franklin Lake, NJ, USA; diluted 1/500) was incubated for 30 min at room temperature. For Hif-1*α* expression analysis, only cells with completely and darkly stained nuclei were regarded as positive. The fraction of Hif-1*α*-positive tumour cells was estimated. For statistical analysis tumours or lymph node metastases with a Hif-1*α*-positive tumour cell fraction >5% were considered positive, else negative.

CA9, DEC-1 and Hif-1*α* analyses were carried out by GVdE and were repeated by the same observer after 1 month. A high reproducibility (*κ*-value >0.95) was found.

### Statistical analysis

Statistical analysis was performed with the SPSS 11.0 software package. A *P*-value <0.05 was considered statistically significant. Normality was tested with a Kolmogorov–Smirnov test assuming normality of data if *P*⩾0.2. If continuous data (e.g. ECP% and TCP%) were normally distributed, correlations were analysed with Pearson's correlation statistics, if not with Spearman's correlation statistics. In case of normal distribution in all subgroups, equality of means was tested with a student *T*-test, if data were not normally distributed, equality of medians was tested with a Mann–Whitney *U*-test. For analysing correlations between categorical variables (e.g. CA9, DEC-1 and Hif-1*α* expression, dichotomised ECP% and TCP%, clinico-pathological variables) the *χ*^2^ test or – when the assumptions of the *χ*^2^ test were not met – the Fisher's exact test, were used. A linear regression analysis with stepwise forward procedure of the ECP% of the lymph node metastases was performed including all angiogenesis, hypoxia and clinico-pathological variables of both primary tumours and lymph node metastases.

## RESULTS

### Angiogenesis and tumour cell proliferation

**Figure 2:**
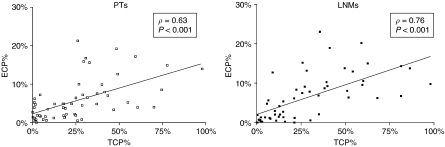
Scatter plot showing a strong correlation between ECP% and TCP% in both primary tumours (left) and lymph node metastases (right). ECP%: endothelial cell proliferation fraction; TCP%: tumour cell proliferation fraction; PT: primary tumour; LNM: lymph node metastasis.

Median ECP% and TCP% in primary tumours and in lymph node metastases were comparable (primary tumours: ECP%=4.02, TCP%=19.54; lymph node metastases: ECP%=5.47, TCP%=21.26; Wilcoxon's signed-ranks test ECP% *P*=0.911; TCP% *P*=0.566). Both in primary tumours and in lymph node metastases, ECP% and TCP% were strongly correlated (Figure 2). Furthermore, ECP% and TCP% in two different lymph node metastases (*N*=15) from the same patient showed a very strong correlation (ECP%: *ρ*=0.80, *P*<0.001; TCP%: *ρ*=0.90, *P*<0.001).

A higher ECP% and TCP% was found in primary tumours with a mixed or expansive growth pattern compared with primary tumours with an infiltrative growth pattern (ECP% *P*=0.03, TCP% *P*=0.08). Median ECP% or TCP% of the primary tumour did not correlate with the presence of a fibrotic focus. No correlation was found between ECP% or TCP% of the lymph node metastases and the growth pattern or presence of a fibrotic focus in the primary tumour. When ECP% and TCP% in the primary tumours and in the lymph node metastases were dichotomised, a high ECP% in the primary tumour was correlated with the presence of a fibrotic focus (*P*=0.05) and with an expansive or mixed growth pattern (*P*=0.05). Furthermore, a high ECP% in the lymph node metastases was correlated with an expansive or mixed growth pattern in the primary tumour (*P*=0.05).

### Hypoxia

**Figure 3:**
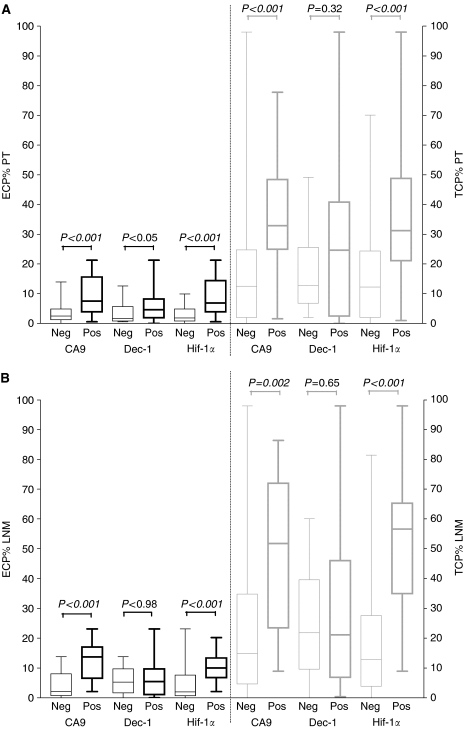
Box and Whiskers plot of ECP% (left, black) and TCP% (right, grey) in CA9, DEC-1 and Hif-1*α*-positive (Pos) and -negative (Neg) primary tumours (**A**) and lymph node metastsases (**B**). Primary tumours and lymph node metastases with CA9 or Hif-1*α* expression had significantly higher ECP% and TCP%. No difference in ECP% or TCP% was found between primary tumours and lymph node metastases with or without DEC-1 expression. PT: primary tumour; LNM: lymph node metatasis; ECP%: endothelial cell proliferation fraction; TCP%: tumour cell proliferation fraction; CA9: carbonic anhydrase 9; Hif-1*α*: hypoxia inducible factor-1*α*; DEC-1: differentiated embro-chondrocyte-expressed gene 1.

CA9, DEC-1 and Hif-1*α* were expressed in, respectively, 38.3, 76.7 and 46.7% of primary tumours. In lymph node metastases, 21.7, 66.7 and 26.7% were positive for, respectively, CA9, DEC-1 and Hif-1*α*. In all, 42.4% of primary tumours did have a fibrotic focus and 25.4, 23.7 and 50.0% of primary tumours had an infiltrative, expansive or mixed growth pattern, respectively. Expressions of CA9 and Hif-1*α* were correlated in primary tumours (*P*<0.001) and in lymph node metastases (*P*=0.005). A trend to a correlation between DEC-1 and CA9 (*P*=0.05) and between DEC-1 and Hif-1*α* expression (*P*=0.05) was found in primary tumours. In lymph node metastases, no correlation between DEC-1 and CA9 (*P*=0.74) or Hif-1*α* (*P*=1.0) was found. Furthermore, when two lymph node metastases from the same patient were compared, a perfect correlation was found for CA9 expression (*P*<0.001). A strong correlation was found for Hif-1*α* (*P*<0.001): in only one case one lymph node metastasis expressed Hif-1*α*, while the other did not. Figure 3 depicts boxplots comparing median ECP% and TCP% in primary tumours (upper part) and lymph node metastases (lower part) positive or negative for, respectively, CA9, DEC-1 and Hif-1*α*. ECP% and TCP% are significantly higher in primary tumours and lymph node metastases expressing CA9 or Hif-1*α*. Furthermore, primary tumours with a fibrotic focus more frequently expressed CA9 (*P*=0.005) or Hif-1*α* (*P*=0.007). Similarly, more CA9 (*P*<0.001) and Hif-1*α* (*P*<0.001) expression was demonstrated in primary tumours with a mixed or expansive growth pattern, compared to primary tumours with an infiltrative growth pattern. No significant correlation was found between the growth pattern of the primary tumour or the presence of a fibrotic focus in the primary tumour and DEC-1 expression in the primary tumour or the expression of CA9, Hif-1*α* and DEC-1 in the lymph node metastases.

### Angiogenesis, hypoxia and clinico-pathological variables

In primary tumours, tumour grade was positively correlated with ECP% (*P*=0.002) and TCP% (*P*<0.001). Her2/neu status positively correlated with ECP% (*P*<0.001), TCP% (*P*=0.004), CA9 (*P*=0.001) and Hif-1*α* expression (*P*=0.005). Estrogen receptor status was negatively correlated with TCP% (*P*=0.003) and CA9 (*P*=0.04) and progesteron receptor status with ECP% (*P*=0.005), TCP% (*P*=0.001), CA9 (*P*<0.001), Hif-1*α* (*P*=0.003) and DEC-1 (*P*=0.02). In lymph node metastases, comparable data were found (data not shown).

### Angiogenesis and hypoxia in lymph node metastases *vs* primary tumours

**Figure 4:**
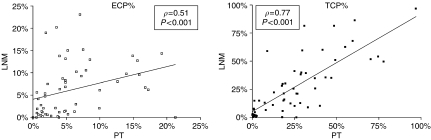
Scatter plot representing the correlation between primary tumours and lymph node metastases for ECP% (left) and TCP% (right). A strong correlation was found for both TCP% and ECP%. ECP%: endothelial cell proliferation fraction; TCP%: tumour cell proliferation fraction; PT: primary tumour; LNM: lymph node metastasis.

A very good correlation was found between TCP% in primary tumours and in lymph node metastases (*ρ*=0.77, *P*<0.001) and between ECP% in primary tumours and in lymph node metastases (*ρ*=0.51, *P*<0.001) (Figure 4). Furthermore, when dichotomised, primary tumours with high ECP% or TCP% had lymph node metastases with high ECP% or TCP%, respectively (ECP% *P*<0.001; TCP% *P*<0.001). Table 2 shows the correlation between primary tumours and lymph node metastases for CA9 and Hif-1*α* expression. A very strong correlation was found both for CA9 (*P*<0.001) and Hif-1*α* (*P*<0.001). For DEC-1 no correlation was found between expression in primary tumours and in lymph node metastases (*P*=0.45). Linear regression analysis revealed that TCP% of the lymph node metastasis (*β*=0.113, *P*<0.001), size of the lymph node metastasis (*β*=0.429, *P*<0.001) and CA9 expression of the lymph node metastasis (*β*=4.088, *P*=0.004) were the most important determinants of ECP% of the lymph node metastases.

## DISCUSSION

In this study, angiogenesis and hypoxia in lymph node metastases of 60 patients with breast cancer were investigated and compared to angiogenesis and hypoxia in the primary tumour. Many different histomorphological methods have been used to assess tumour vascularity and angiogenesis. Endothelial cell proliferation fraction has been described as being the preferred parameter for quantification of angiogenesis (Vermeulen *et al*, 1996, 2002). In contrast to other authors (Vartanian and Weidner, 1994), we demonstrated a strong correlation between TCP% and ECP% both in primary tumours and in lymph node metastases. This correlation suggests that high tumour cell proliferation and increased angiogenesis are linked. High tumour cell proliferation involves increased oxygen consumption, leading to oxygen deficiency and hypoxia-modulated gene expression, including activation of vascular endothelial growth factor and CA9 gene transcription by Hif-1*α* (Forsythe *et al*, 1996; Jiang *et al*, 1997; Maxwell *et al*, 1997; Carmeliet *et al*, 1998). TCP% and ECP% are indeed strongly correlated with CA9 and Hif-1*α* expression. More integrative histological markers of hypoxia-induced stromal tissue remodelling are the growth pattern of the primary tumour and the presence of a fibrotic focus. We previously demonstrated in primary tumours of node-negative breast cancer patients (Colpaert *et al*, 2001) and in skin deposits of breast cancer (Colpaert *et al*, 2003b) that the presence of a fibrotic focus and an expansive growth pattern are correlated with increased angiogenesis and hypoxia. These results are confirmed in this lymph node-positive study population. Furthermore, TCP%, ECP% and hypoxic markers are also correlated with tumour grade and Her2/neu status and inversely correlated with ER and PR status. Existing breast cancer literature is not always straightforward on associations between clinico-pathological data and angiogenesis and hypoxia. Some authors indeed find the same associations (Hansen *et al*, 2000; Chia *et al*, 2001; Bos *et al*, 2003; Koukourakis *et al*, 2003; Giatromanolaki *et al*, 2004; Gruber *et al*, 2004), whereas others do not (Fox *et al*, 1994; Costello *et al*, 1995; Aranda and Laforga, 1996; Schindl *et al*, 2002; Blackwell *et al*, 2004). Differences in study population and in the methodology of angiogenesis quantification make comparison of the results difficult.

The results of this study further suggest that hypoxia is equally important for angiogenesis in primary tumours and lymph node of breast cancer. New breast cancer metastasis models (reviewed in Pantel and Brakenhoff, 2004) emphasise the role of early haematogenous dissemination of tumour cells in breast cancer progression. Nevertheless, since angiogenesis is an important process in tumour progression and metastasis (Folkman, 1986), angiogenesis in lymph node metastases might also contributes to this dissemination. Guidi *et al* (2000) demonstrated that the presence of vascular hotspots and increased microvessel density in axillary lymph node metastases, but not in primary breast cancer, was associated with poor outcome. Increased hypoxia-induced angiogenesis in lymph node metastases might be an explanation for the correlation between CA9 expression in the primary tumour and prognosis, in patients with lymph node-positive breast cancer (Chia *et al*, 2001). Furthermore, the angiogenic potential and growth characteristics of the tumour cells in the primary tumour seem to be preserved during the metastatic process. The clonal theory of the origin of metastases indeed predicts this (Kerbel, 1990). Guidi *et al* (2000) and Edel *et al* (2000) did not find a correlation between the microvessel density and number of proliferating endothelial cells per mm^2^ in primary tumours and in lymph node metastases. In the largest series of 35 ductal breast carcinomas, Arapandoni *et al* demonstrated a correlation between the microvessel score of the primary tumour and the lymph node metastasis, although microvessel score in lymph node metastases was decreased. None of these studies measured hypoxia-marker expression and since different measures were used to quantify angiogenesis, these results are difficult to compare with ours.

Although angiogenic and hypoxic properties of the primary tumour and the lymph node metastases seem to correlate, multiple linear regression analysis withheld only size of the lymph node metastasis, TCP% of the lymph node metastasis and CA9 expression in the lymph node metastasis as factors independently determining the ECP% of the lymph node metastasis. Furthermore, a substantial number of lymph node metastases corresponding to primary tumours positive for CA9 or Hif-1*α*, did not express CA9 or Hif-1*α*. These data suggest that the local tumour microenvironment and the interaction between the characteristics of the metastatic tumour cells and their microenvironment are influencing the angiogenic phenotype of the lymph node metastases. Previous work on different growth patterns at different tumour sites in breast cancer supports these findings. Skin deposits of breast cancer grow according to three different patterns with different angiogenic properties (Colpaert *et al*, 2003b). The expansive growth pattern, with induction of a desmoplastic new stroma, showed a high ECP%, a high microvessel density and a high CA9 expression. In the infiltrative growth pattern much lower values were found. Furthermore, the growth pattern of the skin deposit correlated with the growth pattern of the primary tumour. Breast cancer liver metastases, on the other hand, almost always show a ‘replacement’ growth pattern (Stessels *et al*, 2004): breast cancer cells replace hepatocytes at the tumour/liver interface with conservation of the liver plate architecture and cooption of the sinusoidal vasculature of the liver. Almost no hypoxia and no angiogenic switch is seen. These observations in primary tumours, skin deposits, lymph node metastases and liver metastases of breast cancer suggest an important role for the local, organ-specific microenvironment in determining angiogenesis and hypoxia during breast cancer growth. The liver is, due to its physiological function, a densely vascularised organ and can host metastases that exploit this environment by replacing the hepatocytes and coopting the vasculature. Breast parenchyma, lymph nodes and skin on the other hand are less well vascularised and therefore probably unable to meet the high metabolic demands of a growing malignancy with the pre-existing vasculature. This hypothesis is supported by findings in animal models. Monsky *et al* (2002) described different angiogenic responses when an estrogen-dependent breast tumour line was implanted either into the mammary gland fat pad or into the cranium. Differences in angiogenic and hypoxic properties of different tumour sites might have therapeutic consequences when treatment with antiangiogenic therapy is considered.

In conclusion, this study demonstrated that growth of lymph node metastases in breast cancer, in contrast to metastatic growth in the liver, is angiogenesis dependent and that hypoxia is – at least partially – the driving force for the angiogenesis. Furthermore, the angiogenesis and hypoxia in the lymph node metastases are predicted by the angiogenesis and hypoxia in the primary tumours. These new data, in combination with previous findings, suggest that both the properties of the tumour cells in the primary tumour and the microenvironment of tumour growth in breast cancer are important in determining the angiogenic and hypoxic properties. In the case of the lymph nodes it may be that the oncogene programme is the main driver for generating hypoxia and induction of angiogenesis. Evidence for this is the strong association between Her2/neu with Hif-1*α*. Variation in angiogenesis and hypoxia dependent on the metastatic site have important implications for anti-Hif-1*α* and antiangiogenic therapies.

## Figures and Tables

**Table 1:** Clinico-pathological data of patients included

	**Clinico-pathological data (*N*=60)**
Mean age (years)	59.2 (25.3–84.4)
*T status* a	
T1	17
T2	33
T3	5
T4	5
	
*N status* a	
N1	28
N2	19
N3	13
	
*Histological type PT*	
Ductal	48
Lobular	10
Special type	2
	
*Tumour grade PT* b	
I	18
II	25
III	17
	
*ER status PT*	
Negative	17
Positive	43
	
*PR status PT*	
Negative	27
Positive	33
	
*p53 status PT*	
Negative	40
Positive	20
	
*Her2/neu status PT*	
Negative	48
Positive	12

PT=primary tumour; ER=estrogen receptor; PR=progesteron receptor.

a T and N status were assigned according to the TNM classification of the American Joint Committee on Cancer (Green *et al*, 2002).

b Tumours were histologically graded according to the Nottingham modification of the Bloom and Richardson histological grading scheme (Bloom and Richardson, 1957; Elston, 1987).

**Table 2:** Correlation between hypoxia parameters in primary tumours and in lymph node metastases

**CA9 *P*<0.001 Hif-1*α* *P*<0.001**	**LNM**				
	**CA9 negative**	**CA9 positive**		**Hif-1*α* negative**	**Hif-1*α* positive**
*PT*					
CA9 negative	31	1	Hif-1*α* negative	26	1
CA9 positive	11	12	Hif-1*α* positive	13	15

(PT: primary tumour, LNM: lymph node metastasis, CA9: carbonic anhydrase 9, Hif-1*α*: hypoxia inducible factor-1*α*).

CA9 and Hif-1*α* expression in primary tumours and in lymph node metastases correlated (CA9 and Hif-1*α* *P*<0.001).
